# Targeting CD73 and correcting adenosinergic signaling in critically ill patients

**DOI:** 10.3389/fphar.2025.1601481

**Published:** 2026-01-12

**Authors:** Justin Mark Lunderberg, Alexander James Spicer, Jessica Cassavaugh, Juho Jalkanen, György Haskó, Simon C. Robson

**Affiliations:** 1 Department of Anesthesiology, Center for Inflammation Research, Beth Israel Deaconess Medical Center and Harvard Medical School, Boston, MA, United States; 2 Faron Pharmaceuticals Ltd., Turku, Finland; 3 Department of Anesthesiology, Columbia University, New York, NY, United States

**Keywords:** adenosine, ATP, CD39, CD73, ischemia and reperfusion, purinergic, sepsis

## Abstract

The concept of the intensive care unit (ICU) was developed around 70 years ago, and this has become essential for caring for a hospital’s sickest patients. A commonality for many patients’ critical illness is the systemic inflammatory state with multiple-organ injury precipitated by infectious causes and/or other pathophysiologic insults. Therapeutic modalities to address these complications remain unclear, and there are no FDA-approved drugs to treat these often-devastating clinical situations. Clinical deterioration may be associated with the release of “damage-associated molecular patterns” (DAMPs), such as extracellular adenosine triphosphate (eATP), from stressed and dying cells. Pharmacological dosing or boosting of CD73, an ectoenzyme that can convert pro-inflammatory adenosine monophosphate (AMP) to anti-inflammatory adenosine, in this setting of critical illness has been shown to have a survival benefit with decreased time in the hospital and ICU. Whether there are clinical benefits of extracellular nucleotide (eATP) scavenging over adenosine generation within the setting of inflammatory diseases remains unclear. Upcoming pre-clinical developments testing soluble forms of CD39 to hydrolyze eATP to AMP in sepsis and following cardiac surgery should clarify this question. We conclude by suggesting that exogenous CD39, CD73, and/or other ectonucleotidases may provide therapeutic benefits in critically ill patients.

## Graphical Abstract

**Figure:**
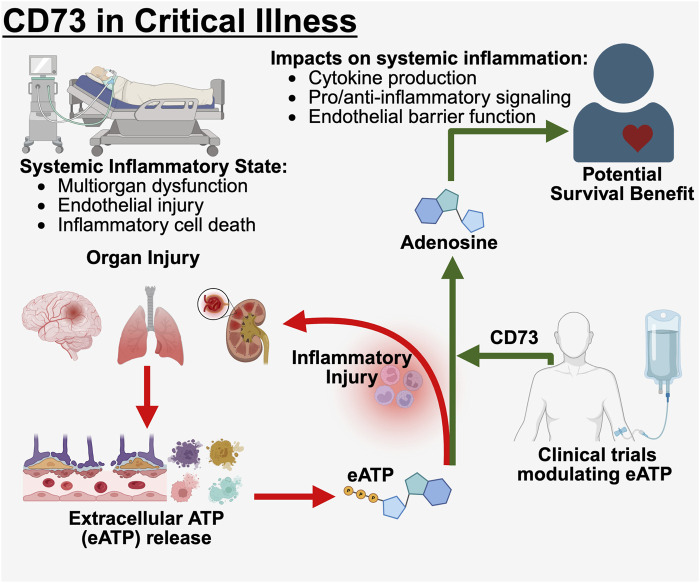

## Introduction

The ideas underpinning critical care were initially adopted during the 1952–1953 Copenhagen polio epidemic, where medical practitioners found that the assisted ventilation of polio patients decreased their mortality rate by half (Lassen, 1953). While unique life-saving supportive care can be provided within an intensive care unit (ICU), prior observation and the recent coronavirus disease 2019 (COVID-19) pandemic have highlighted suboptimal quality-adjusted life outcomes following hospitalization (Cuthbertson et al., 2010; Halvorsen et al., 2023). A table of abbreviations is presented in Table 1. Recent advances in vaccine technology provided useful tools to blunt the COVID-19 public health crisis and pandemic; however, similar advances in innovative therapeutics for critically ill patients to alleviate morbidity and mortality have not been realized. In part, this may be due to the inherent challenges of the ICU having heterogeneous patient populations being subject to syndromic-type illness arising from multiple disease states (Santacruz et al., 2019).

**TABLE 1:** Abbreviations and expanded definitions used within this work.

Abbreviation/Acronym	Expanded definition
ACDC	Arterial calcification due to deficiency in CD73
ADA	Adenosine deaminase
ADP	Adenosine diphosphate
AKI	Acute kidney injury
AMP	Adenosine monophosphate
AP	Alkaline phosphatase
ARDS	Acute respiratory distress syndrome
ATP	Adenosine triphosphate
cAMP	Cyclic adenosine monophosphate
CD39	Ectonucleoside triphosphate diphosphohydrolase-1
CD73	Ecto-5′-nucleotidase
COPD	Chronic obstructive pulmonary disease
COVID-19	Coronavirus disease 2019
CRP	C-reactive protein
DAMP	Damage-associated molecular pattern
eATP	Extracellular adenosine triphosphate
eAMP	Extracellular adenosine monophosphate
HMG-CoA	3-hydroxy-3-methylglutaryl-coenzyme A
HIF	Hypoxia-inducible transcription factor
HRE	HIF-response element
ICAM-1	Intercellular adhesion molecule-1
ICU	Intensive care unit
IFN	Interferon
KO	Knockout
LDL	Low-density lipoprotein
MS	Multiple sclerosis
NSCLC	Non-small cell lung cancer
NAD	Nicotinamide adenine dinucleotide
NFAT	Nuclear Factor of Activated T-cell
PD-1	Programmed cell death protein 1
SLE	Systemic lupus erythematosus
SOFA	Sequential organ failure assessment
VACM-1	Vascular cell adhesion molecule-1

In this review, we propose that modulating fundamental immune signalling pathways and ameliorating deleterious inflammatory responses may help patients recover from critical illness and offer protection from end-organ injury. The manuscript will review preclinical and clinical evidence for indirectly and directly targeting membrane ectonucleotidases, specifically ecto-5′-nucleotidase (CD73), and we integrate the discussion to include a model for future investigation of adenosine triphosphate- (ATP)-metabolite modulation followed by glucocorticoid administration within infectious inflammatory injury states.

Methodologically, clinicaltrials.gov and EudraCT were searched for the terms CD39, CD73, and adenosine in association with registered trials and Pubmed was queried for studies performed between 1990 and 2024 with the terms CD73, CD39, critical care, sepsis, and ARDS.

Broadly, purines, or the class of molecules that contain the product of the fusion of a pyrimidine and imidazole ring, are the most widely occurring nitrogen-containing naturally occurring heterocycles. Members of this class are used as an energy currency through the serial phosphorylation of adenosine-to-adenosine triphosphate, with these same molecules also being used in extracellular signalling pathways (Linden et al., 2019; Burnstock, 2020; Eltzschig et al., 2013). Identifying effects of the purinome, encompassing synthesis and metabolism of purines, modulation of receptors, and downstream signalling pathways, are all important to achieve a greater understanding of human health and disease (Idzko et al., 2014; Huang et al., 2021; Eltzschig et al., 2013).

Herein, we describe the specific effects of pharmacologic modulation of CD73, a cell-surface enzyme that metabolizes adenosine monophosphate (AMP) to adenosine and was originally identified as an organ-protective endothelial molecule in states of hypoxia and inflammation (Thompson et al., 2004; Eltzschig et al., 2013; Eltzschig, 2013; Le et al., 2019). The extracellular AMP (eAMP) moity is typically formed by the action of ectonucleoside triphosphate diphosphohydrolase-1 (CD39), serially catalysing the hydrolysis of extracellular ATP (eATP) to eAMP (Allard et al., 2017). We further describe recent work inducing CD73 expression through interferon beta-1a administration that has indicated protective effects in patients presenting for emergent surgery for a ruptured abdominal aortic aneurysm and other recent trials in the field (Hakovirta et al., 2022). Such upregulation of CD73 activity or eventual use of soluble forms of CD73 may help address endothelial vasculature dysfunction, a common pathology for the largely heterogeneous critically ill patient population (Vincent et al., 2021; Cribbs et al., 2008).

It should also be noted that gene mutations in CD73 and a lack of the associated AMPase function have been associated with the clinical development of severe calcification in the large arteries of adults (St Hilaire et al., 2011). This inherited disorder, arterial calcification due to CD73 deficiency (ACDC) indicates protective functions of CD73 in the vasculature over the long term. Additional clinical benefits of extracellular eATP scavenging vs. increased adenosine generation within the setting of inflammatory diseases remain unclear. Pre-clinical developments testing soluble forms of CD39 to boost hydrolysis of eATP to AMP may clarify future use of therapies targeting purinergic responses in the ICU, as with sepsis and complications following cardiac surgery.

## Critical illness and inflammatory purinergic signaling

Modulation of purinergic signaling pathways may be a tool to address the damaging effects of systemic inflammation in critical illness. Systemic inflammation and multi-organ failure can be precipitated by multiple infectious and non-infectious disease states (Cauwels et al., 2014). Changes in care, including ventilatory strategies within acute respiratory distress syndrome (ARDS), bundled actions decreasing central-line infections, and goal-directed responses to sepsis, have been associated with improved outcomes for patients; yet, there have not been similar improvements seen in critically ill patients undergoing trials of corticosteroids, aspirin, statins, growth factors, activated protein C, stem cells, as well as vitamins C and D (Matthay et al., 2017; François et al., 2016; Vincent and Creteur, 2015). The unsuccessful trials and the broad range of etiologies precipitating systemic inflammation have led to the concern that a unifying disease-modifying factor is not present.

**FIGURE 1:**
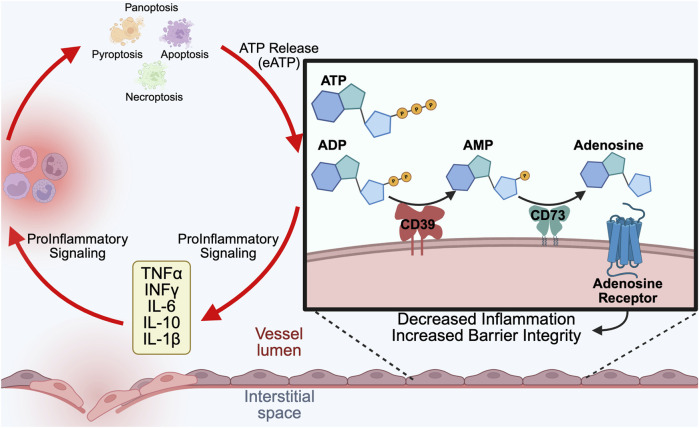
CD39 and CD73 control pro-inflammatory eATP signaling and promote adenosine-associated anti-inflammatory effects and barrier integrity. On the upper left of the graphic, inflammatory programmed cell death pathways lead to the release of extracellular ATP (eATP). Downstream signaling causes the release of pro-inflammatory cytokines, causing impairment of barrier integrity and is associated with further apoptosis and necrosis. This cycle is modulated by CD39-catabolized degradation of eATP to ADP and then AMP and CD73-dependent formation of adenosine from AMP (right side of figure). Adenosine signaling decreases inflammatory signaling and maintains barrier integrity.

In systemic inflammation associated with multi-organ failure, a common feature independent of the causative insult includes an increase in vascular permeability followed by fluid and leukocyte extravasation (Duan et al., 2017; Vincent et al., 2021). This process is associated with stressed and dying cells releasing ATP, a putative damage associated molecular pattern (DAMP), into circulation as eATP, where it acts as a key pro-inflammatory mediator (Gordon, 1986; Cauwels et al., 2014). Subsequently, eATP signaling causes downstream systemic induction of cytokines associated with mitochondrial damage and the initiation of apoptotic cell death pathways (Cauwels et al., 2014). An illustration of this signaling pathway highlighting the activity of CD39 and CD73 is provided in Figure 1. The ability to metabolize circulating eATP into anti-inflammatory adenosine is a key adaptation in withstanding inflammatory insults (Salmi and Jalkanen, 2005; Eltzschig and Carmeliet, 2011).

The canonical enzyme cascade creating eAMP involves CD39, as identified here (Kaczmarek et al., 1996). However, ENPP1, also known as CD203a, can generate eAMP from the metabolism of nicotinamide adenine dinucleotide (NAD) and adenosine diphosphate- (ADP)-ribose with the associated links to CD73 (Stagg et al., 2023).

## CD73: a link between pro-inflammatory eATP and anti-inflammatory adenosine signaling

We note the pro-inflammatory signaling cascaded precipitated by eATP and yet a degradation product of this molecule, adenosine, is involved in anti-inflammatory signaling. This product can be formed by the sequential action of CD39 catalyzing the degradation of eATP to AMP and then CD73 catalyzing phosphate removal from AMP to form adenosine. CD73 is expressed on a wide range of cell types, including hemopoietic lineages and endothelial cells, as well as subsets of epithelial cells, where it is anchored to the cell surface (Zhong et al., 2021). Within vascular endothelial and epithelial layers, CD73 preserves barrier function as a function of anti-inflammatory adenosine production (Figure 1) (Yegutkin, 2008; Synnestvedt et al., 2002; Antonioli et al., 2013). Adenosine inhibits the function of E-selectin, vascular cell adhesion molecule 1 (VCAM-1), and intercellular adhesion molecule-1 (ICAM-1), as well as the expression of integrins and the release of some cytokines, resulting in decreased neutrophil adhesion to vascular endothelial cells and subsequent extravasation into tissue (Grünewald and Ridley, 2010). Furthermore, adenosine induces arterial dilatation, increasing tissue perfusion, which can counteract localized ischemia (Sotnikov and Louis, 2010).

Adenosine has four cognate G-protein-coupled receptors: A_1_, A_2A_, A_2B_, and A_3_. Signaling initiated by the A_1_ and A_3_ receptors inhibits cyclic adenosine monophosphate (cAMP) accumulation, whereas A_2A_ and A_2B_ receptor signaling induces cAMP accumulation. An understanding of the anti-inflammatory effects of adenosine A_2A_ receptor-mediated anti-inflammatory mechanisms is predicated on pioneering work by Sitkovsky and colleagues (Ohta et al., 2006; Ohta and Sitkovsky, 2001). Immune cell expression and downstream consequences of adenosine receptor activation all appear to be cell-type specific (Zhang et al., 2022). Receptor affinities towards adenosine vary, with A_1_ and A_2A_ exhibiting greater affinity; notably, the affinity of A_2B_ towards adenosine is much lower, and A_3_ is species-dependent (Cronstein and Sitkovsky, 2017).

Five to fifteen percent of blood lymphocytes express CD73. Notably, CD73 is present in regulatory T cells, which are a cell population involved in the resolution of cell injury and the dampening of an immune response (Kobie et al., 2006; Deaglio et al., 2007; Ehrentraut et al., 2013; Borg et al., 2017). In addition, CD19-positive B cells also express CD73 (Saze et al., 2013). The immunosuppressive function of the regulatory T cells and B cells is mediated by the CD73-derived adenosine, as it directly inhibits the proliferation and activation of effector T cells (Kepp et al., 2017). Adenosine also affects the polarization of macrophages (Li and Okusa, 2010; Kayagaki et al., 2011). Adenosine, particularly through A_2A_ receptor signaling, drives the polarization of macrophages to an M2 immunosuppressive phenotype, which has been proposed to play a role in attenuating inflammation (Chávez-Galán et al., 2015; Csóka et al., 2012; Koscsó et al., 2013).

This A_2A_ receptor-mediated signaling has also been implicated in anti-inflammatory effects, including the inhibition of neutrophil adhesion and neutrophil degranulation (Barletta et al., 2012). In contrast, proinflammatory eATP induces a macrophage M1 phenotype, which is associated with clearing invading organisms, even to the point of self-injury (Ley, 2017). M1 macrophages further promote their polarization in a feed-forward loop through the induction of a Th1 T cell response, enhancing M1 polarization with IFN-γ (Green et al., 2013; Unsinger et al., 2010). Cell-specific differences in the adenosine receptor expression profiles and downstream adenosine signaling can lead to difficulty in precisely predicting the effects of adenosine modulation at the organ level.

## Transporter and degradation machinery impact on extracellular adenosine flux

In addition to adenosine signaling through extracellular receptor interactions, this nucleoside can also be transported across the cell membrane through nucleoside transporters. Although the immunologic effects of adenosine receptor stimulation have been well studied, the impact of adenosine transporters on intracellular responses has remained somewhat unexplored. Previous studies have shown that cellular adenosine uptake occurs via ENT1 and modulates intracellular metabolism in both T cells and cancer cells (Apasov and Sitkovsky, 1999; Spychala, 2000; Losenkova et al., 2020). The impact of drugs that inhibit adenosine uptake, primarily by ENT1 blockade, appears to be immunostimulatory, with recent studies revealing increased T cell responses in the setting of ENT1 deficiency or blockade (Allard et al., 2025). Hence, the targeting of ENT1 via pharmacological blockade results in the accumulation of extracellular adenosine, resulting in potential signaling events via activation of the A_2A_ receptor as well as modulation of intracellular nucleotide metabolism and bioenergetics. Pharmacologic manipulation of adenosine uptake can be performed by the drug dipyridamole, though it has multi-functional effects with the breakdown of cAMP as well as the inhibition of adenosine uptake by ENT1. Research has further shown that dipyridamole inhibits the Nuclear Factor of Activated T-cell (NFAT) interactions without affecting overall calcineurin phosphatase activity to negatively impact T-cell activation and proliferation in systemic lupus erythematosus (SLE) (Kyttaris et al., 2011).

Several studies have explored the inhibitory effects of dipyridamole on T-lymphocyte proliferation and suggest the overall effect is one of weak immunosuppression. Massaia et al. have previously demonstrated that dipyridamole suppresses the generation of alloreactive cytotoxic T lymphocytes in a dose-dependent manner indicating that dipyridamole prevents the initial activation step of the lytic program in T-cells following allogeneic or interleukin-2 stimulation (Massaia et al., 1991). Of note, such opposing mechanisms of this drug on adenosine-mediated immunosuppression vs. potential for cellular activation are not mutually exclusive, as these may be selectively present in various T cell subtypes. Indeed, both may be concurrently operational in regulating and fine-tuning T cell responses.

Adenosine deaminase (ADA) functions to degrade adenosine and 2′-deoxyadenosine to inosine and 2′-deoxyinosine respectively (Whitmore and Gaspar, 2016). There are two ADA enzymes, ADA1 and ADA2, with ADA1 genetic deficiency being the most common cause of severe combined immunodeficiency (SCID) due to buildup of purine metabolites primarily impacting lymphatic cells and ADA2 deficiency precipitating a complex phenotype of inflammation and immunodeficiency (Ehlers and Meyts, 2025; Sauer et al., 2012). ADA2 deficiency was more recently classified in 2014, with the complex phenotype including early onset stroke, vasculitis, and immunodeficiency (Lee et al., 2023). ADA2 is preferentially expressed in immune cells and is released into the extracellular space where there can be significant increases in activity within inflammatory disease, including tuberculosis and SLE [reviewed in, (Ehlers and Meyts, 2025),]. ADA2 deficiency would presumably lead towards an immunosuppressive state given increases in extracellular adenosine yet the opposite phenotype is observed, providing an opportunity for further research into the enzymes effect on localized purinergic flux. ADA inhibition can be achieved through the use of the drug pentostatin, a purine analogue, and is clinically impactful in the extreme sense of treating hairy cell leukemia where the drug is particularly toxic to lymphocytes (Johnston, 2011). We suggest there may be deleterious and possible off-target manifestations following the pharmacologic manipulation of global ADA activity (ADA1 and ADA2) in critically ill patients. This consideration is based on the observed role of ADA2 in interferon release, as modulated by TLR9 responses to DNA, and also the development of clinically severe vasculitis observed following ADA2 mutations (Dong et al., 2024; Moens et al., 2019).We do note that low-dose deoxycoformycin, *i.e.*, pentostatin, a transition state analogue drug blocking both ADA1 and ADA2, has been administered in mouse experimental models of atherosclerosis with an observable benefit on plaque evolution; at this dosing it did not have systemic toxicity with 90% inhibition of ecto-ADA activity (Kutryb-Zajac et al., 2016). Therefore, targeted pharmacologic ADA inhibition within states of inflammation may be feasible in the future once further work has been done on the structure-function relationships of ADA1 and ADA2, which has resulted in the development of new, more specific, and safer drug candidates.

## Clinical developments: CD73 inhibition as an oncologic and COVID-19 drug target

Typically outside of the ICU, CD73 has emerged as an attractive oncologic therapeutic target because the enzyme affects the tumor microenvironment, with tumor CD73 upregulation establishing an anti-inflammatory milieu through the generation of adenosine, promoting tumor growth under hypoxic conditions (Harvey et al., 2020; Roh et al., 2020). While not upregulated on all tumors, oncologic lineages with increased CD73 expression have a degree of protection against eATP, proinflammatory cytokines, and tumor-killing immune cells; this, in turn, conveys a worse prognosis for the patient with some forms of cancer (Roh et al., 2020). CD73 blockade via a monoclonal antibody or a small molecule inhibitor treatment may enhance the effectiveness of current immunotherapies (Ghalamfarsa et al., 2019).

Currently, the CD73 antibody inhibitor furthest along in clinical development is Oleclumab, which is currently in a phase III trial (PACIFIC-9, NCT05221840) in combination with durvalumab, a programmed cell death protein 1 (PD-1) inhibitor, for non-small cell lung cancer (NSCLC) (Barlesi et al., 2024). Trial work commenced following encouraging data from the COAST trial in NSCLC (COAST, NCT03822351) (Herbst et al., 2022).

The potential for an anti-CD73 monoclonal antibody to benefit patients with COVID-19 infection was assessed during the pandemic. Mupadolimab (CPI-006, CORVUS Pharmaceuticals), a humanized monoclonal antibody directed against CD73, has a proposed dual mechanism of activating B cells and inhibiting the production of adenosine (Miller et al., 2022). Downstream signaling following antibody-mediated engagement has been described, but there has not been a natural ligand for this signaling identified to this point (Da et al., 2022). Regardless, Mupadolimab was investigated in the context of hospitalized patients with mild or moderate COVID-19 disease, *i.e.,* with SpO2≥94% without the addition of supplemental oxygen, and antibody administration was notable for potentially inducing an enhanced adaptive antiviral response (NCT04464395) (America, 2021). A phase 3 follow-up trial was discontinued with 40 patients recruited due to the decreasing incidence of COVID-19 (NCT04734873) (Miller et al., 2021).

## Pre-clinical developments: adenosine signaling manipulation as a target in systemic inflammation

In contrast to the primarily oncologic drug development focused on CD73 inhibition, CD73 induction or the theoretical possibility of supplementation may be beneficial within states of systemic inflammation through improving the maintenance of vascular integrity and providing protection against multi-organ failure (Roh et al., 2020; Cauwels et al., 2014). Over the past 2 decades, a large body of literature revealed extracellular nucleotide metabolism [reviewed in (Gordon, 1986; Dale and Frenguelli, 2009; Dale, 2021)] is a key mechanism in the prevention of induced vascular leakage in mouse models (Eckle et al., 2007b; Grenz et al., 2007; Kiss et al., 2007; Cai et al., 2013).

Therapeutics for inflammatory disorders involving wound healing, ischemia, and arthritis have been successfully developed by targeting the purinergic signaling pathways via ATP receptor antagonists (Haskó et al., 2008). Moreover, adenosinergic modulation has further potential for organ protection in hypoxia (Haskó et al., 2011).

**FIGURE 2:**
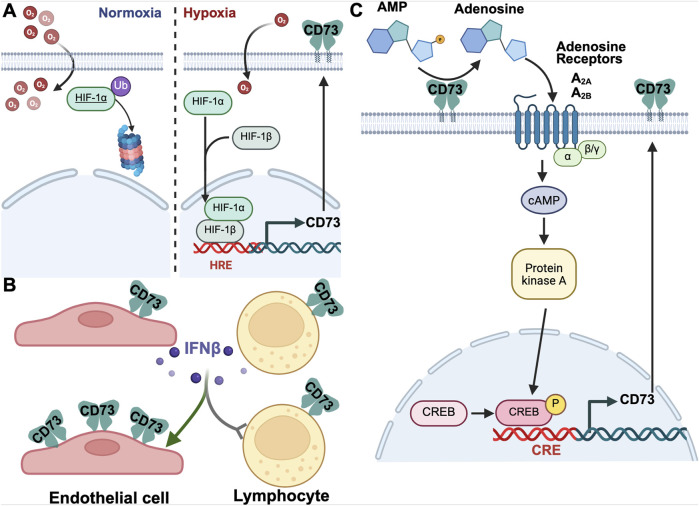
Modes of CD73 induction. **(A)** CD73 is induced under hypoxic conditions, at least in part through the HIF pathway. Under normoxic conditions, (left), HIF1α is ubiquinated and rapidly degraded. Under hypoxic conditions, (right), HIF1α is not degraded, associates with HIF1β, and translocates to the nucleus. Here, it enhances the gene expression of CD73, among many proteins, through a hypoxia response element (HRE). **(B)** Interferon β induces the production of CD73 in endothelial cells but not lymphocytes. **(C)** CD73 expression is induced in an autocrine manner with CD73-derived adenosine signaling through adenosine receptors (A_2A_ and A_2B_), causing an increase in cyclic AMP with subsequent signaling culminating in increased CD73 expression.

There is a tightly connected interplay between hypoxia and inflammation. Mammals have an adaptive response to hypoxia that includes oxygen-sensing prolyl hydroxylases modifying hypoxia-inducible transcription factor (HIF) (Epstein et al., 2001). This subsequently activates, among others, the transcription of a range of purinergic metabolizing and receptor genes via the HIF-response element (HRE) in their promoter (Figure 2A) (Figarella et al., 2024; Eltzschig and Carmeliet, 2011; Lee et al., 2019). The CD73 gene promoter also contains a cAMP response element, through which CD73-derived adenosine triggers the expression of CD73 in the positive feedback loop (Figure 2C) (Narravula et al., 2000; Hansen et al., 1995).

While many cell types express CD73, expression is differentially regulated and cell-type specific. Type 1 interferons (IFNs), IFN-alpha and IFN-beta upregulate the expression and enzymatic activity of CD73 on the vascular endothelium without influencing lymphocytic expression of CD73 (Figure 2B) (Niemela et al., 2004). In a mouse CD73 −/− model, a lack of CD73 activity correlates with decreased expression of type 1 IFN and greater pathologic severity in models of experimental colitis; it is unclear whether this change is caused by decreased adenosine production or the accumulation of precursor substrates (Sotnikov and Louis, 2010). Regardless, the administration of type 1 IFNs, including IFN-alpha in urothelial carcinoma and IFN-beta in multiple sclerosis (MS), is associated with increased vascular CD73 expression without a change in CD73 expression on lymphocytes or carcinoma cells (Airas et al., 2007; Niemela et al., 2008; Chambers and Matosevic, 2019; Niemela et al., 2004). Therefore, type I IFN administration may offer benefits by potentiating vascular CD73 expression in settings of intravascular inflammation or hypoxic insults.

## Pre-clinical developments: experimental models in support of CD73-based therapy within inflammatory states

The role of CD73 within adenosinergic signaling has been explored using knockout (KO) mice in multiple disease models (Zernecke et al., 2006; Tsukamoto et al., 2012; Haskó et al., 2011; Chrobak et al., 2015; Kiss et al., 2007; Grenz et al., 2007). CD73 null mice are minimally impacted by their genetic mutation at baseline, yet exhibit greater symptom severity in disease models for acute lung injury and sepsis, presumably due to increased vascular leakage and the lack of immunosuppression (Kiss et al., 2007; Ehrentraut et al., 2013; Haskó et al., 2011). Type 1 IFNs upregulate CD73 expression, and while IFN-beta has multiple downstream targets, induction of CD73 upregulation by IFN-beta has a positive impact in a murine model of acute lung injury, an effect dependent on mouse CD73 expression (Kiss et al., 2007). Further, acute lipopolysaccharide-induced lung injury, organ injury associated with hemorrhagic shock, and mechanical-ventilation-induced acute lung injury are ameliorated in mouse models by soluble CD73 administration (Eckle et al., 2007a; Ehrentraut et al., 2013; Kelestemur et al., 2023). Correlating with cellular and clinical data, adenosine production can be detrimental in murine cancer models (Zhang, 2012) with multiple cancer cell lines, including B16 melanoma, MC38 colon cancer, EG7 lymphoma, and AT-3 mammary tumors, growing more slowly in CD73 KO mice compared to wild-type mice (Yegutkin et al., 2011; Stagg et al., 2011).

## Clinical developments: CD73 expression as a prognostic tool in inflammatory disease

CD73 is upregulated in inflammatory and ischemic conditions as a protective response, and the quantification of changes in its expression and activity may have utility as a prognostic tool. The quantity of soluble CD73 and associated enzymatic activity can be measured in blood and tissue samples. Early studies, defined below, focused on patients with acute pancreatitis, post-operative cardiac surgery, and multiple sclerosis, all potentially severe sterile inflammatory states, as well as more recently in COVID-19 disease (Maksimow et al., 2014; Persson et al., 2018; Niemela et al., 2008; Rud et al., 2023).

ptIn a population of 161 patients with acute pancreatitis on hospital admission, soluble CD73 concentration, CD73 enzymatic activity, and leukocyte CD73 mRNA were inversely correlated with disease severity (Maksimow et al., 2014). Furthermore, low soluble CD73 activity at admission was a more accurate predictor than C-reactive protein (CRP) or creatinine change for the development of severe pancreatitis. Serial measurements of 85 infants who underwent major cardiovascular surgery in their first 120 days of life showed increased levels of postoperative serum CD73. There was also an inverse correlation between the CD73 levels at the time of rewarming during cardiopulmonary bypass and the consequent ionotropic support requirements (Persson et al., 2018). Thus far, the evidence does not support the extension of this predictive factor to infection-associated inflammatory states, as within a population with severe sepsis or septic shock, no associations were identified between soluble CD73 levels and the development of acute kidney injury (AKI) or 90-day mortality (Vaara et al., 2016).

Extending to the active induction of CD73 expression, within a small study analyzing IFN-beta’s effect on newly diagnosed MS patients, 10 out of 11 patients showed increases in their levels of soluble CD73, which was also associated with a decrease in MS relapse rate (Niemela et al., 2008). These studies in sterile inflammatory states reveal that quantifying soluble CD73 may have prognostic value in identifying individuals who are not responding appropriately to infectious inflammatory states.

Decreased circulating CD73 portends worse outcomes in COVID-19. Within a 28-patient subset of patients with mild to severe COVID-19 infection, the degree of reduction in CD73 expression directly correlated with negative outcomes, including ICU admission, need for mechanical ventilation, and hospital length of stay (Rud et al., 2023). Further changes in adenosine signaling pathways beyond CD73 expression may impact the response to COVID-19, including immunologic dysfunction and an impaired T-cell response. One of us (SCR) found within severe COVID-19 disease an elevation in T-cell CD39 expression, a marker of immune exhaustion (Wang et al., 2021). These initial attempts at using CD73 as a prognostic tool are limited by the impact of timing and location of adenosine signaling in response to a pathologic insult.

## Clinical developments in modulating extracellular adenosine production within inflammatory states: alkaline phosphatase

Clinical therapeutic purinergic development has included exogenous alkaline phosphatase (AP) supplementation by AM-Pharma BV (Utrecht, Netherlands), IV IFN-beta by Faron Pharmaceuticals Ltd. (Turku, Finland), inhaled IFN-beta by Synairgen Ltd. (Southampton, United Kingdom), and soluble CD39 by Novartis AG (Basel, Switzerland). Subcutaneous IFN-beta (Rebif, Merck KGaA, Darmstadt, Germany) was investigated in COVID-19 pandemic trials (ACTT-3 and SOLIDARITY) (Beigel et al., 2020; Kalil et al., 2021; Consortium, 2022). A curated list of registered clinical trials discussed here is summarized in Table 2.

**TABLE 2:** Curated list of registered clinical trials focused on CD73 or AMP degradation mentioned within text.

Drug mechanism	Trial	Major findings/Trial description	References (if available)
CD73 inhibition for oncologic endpoint or COVID-19 treatment	NCT03822351; COAST; NCT05221840; PACIFIC-9	Oleclumab (anti-CD73 mAb) enhanced progression free survival in combination with durvalumab in patients with unresectable stage III non-small-cell lung cancer. PACIFIC-9 continues as subsequently active Phase III trial	Herbst et al. (2022), Barlesi et al. (2024)
	NCT04464395	Potential enhanced adaptive antiviral response of Mupadolimab in mild-moderate COVID-19	​
	NCT04734873	Mupadolimab in mild or moderate COVID-19 disease, stopped recruiting due to decreasing incidence of COVID-19	Miller et al. (2021)
Interferon-Beta administration for CD73 induction	NCT00789685	IFN-beta increases CD73 expression and reduces 28-day mortality in ARDS	Bellingan et al. (2014)
	NCT02622724; INTEREST	IFN-beta in moderate and severe ARDS did not identify difference in 28d composite endpoint (including mortality and number of ventilator free days)	Ranieri et al. (2020)
	NCT03119701; INFORAAA	Patients with high level of serum CD73 associated with survival (P = 0.001) whereas the use of glucocorticoids and the presence of IFN beta-1a neutralizing antibodies associated with a poor CD73 response and survival	Hakovirta et al. (2022)
	NCT04315948; SOLIDARITY	Interferon administration within COVID-19 discontinued for futility	Consortium (2022)
	NCT04385095	Phase II trial with inhaled IFN beta-1a increasing potential recovery from COVID-19	Monk et al. (2021)
	NCT04732949; SPRINTER	Inhaled IFN-beta within phase III for covid-19 with no changes in recovery time and time to hospital discharge	Monk et al. (2023)
	NCT04492475; ACTT-3	Interferon beta-1a plus remdesivir was not superior to remdesivir alone in hospitalized patients with COVID-19 pneumonia	Kalil et al. (2021)
Alkaline phosphatase- or CD39-induced eATP hydrolysis	APPIRED II	Phase II study of bovine AP in cardiac surgery population; without TNF-alpha lowering activity identified	Keizer et al. (2021)
	NCT02182440	Alkaline phosphatase treatment did not improve renal function in patients with sepsis-associated AKI	Pickkers et al. (2018)
	NCT04411472; REVIVAL	Phase III study of AP supplementation for sepsis and COVID-19 associated AKI; halted for pre-specified futility measures	Pickkers et al. (2024)
	NCT05524051	Recombinant soluble CD39 in ongoing trial for AKI associated with major cardiac surgery	​
	NCT05996835	Recombinant soluble CD39 in ongoing trial for AKI associated with sepsis	​
Statin-supplementation within ARDS	HARP-2	Simvastatin did not improve clinical outcomes in population with ARDS, *post hoc* analysis with benefit in hyperinflammatory ARDS.	Calfee et al. (2018), McAuley et al. (2014)

Supplementation with AP, an enzyme that hydrolyses the phosphoanhydride bonds attaching phosphate moieties in eATP, showed early promising results in preventing AKI associated with sepsis, burns, solid organ transplantation, major cardiovascular surgery, and chronic inflammatory conditions (Pickkers et al., 2012; Peters et al., 2015; Lukas et al., 2010; Poschner et al., 2021). However, phase II and III studies did not recapitulate these positive early results. Indeed, the initial phase II study of recombinant AP supplementation in sepsis revealed no positive impact on renal function, despite an observed mortality benefit (Pickkers et al., 2018). Phase III studies of AP supplementation were terminated for both sepsis-associated AKI and COVID-19-associated AKI (NCT04411472) after meeting pre-specified futility thresholds (Pickkers et al., 2024). As discussed by Pickkers and colleagues, it is plausible that the signal identified in early phase studies was a type I error, but the lack of signal within Phase III studies could also be impacted by changes in the study population and timing of patient enrollment within their disease course (Pickkers et al., 2024). There are no positive results for AP supplementation in post-cardiac surgery patient populations (Keizer et al., 2021; Kats et al., 2009).

## Clinical developments in modulating extracellular adenosine production within inflammatory states: induction of CD73

Work within ARDS was predicated on the understanding that IFN-beta upregulates lung endothelial CD73 (Bellingan et al., 2014). Following preclinical studies, an open-label study of ARDS patients in eight United Kingdom centers with IV IFN-beta formulations was performed as a phase I and II study (NCT00789685; EudraCT: 2014-005260-15) (Bellingan et al., 2014). Increased levels of soluble CD73 occurred at a dose of 10 μg of IFN-beta per day; phase II patients received this dose for six consecutive days following a diagnosis of ARDS. The 28-day mortality in the treatment group was 8% compared with 32% in the control group, and there was a decreased requirement for renal and vasopressor support in the treatment group (Bellingan et al., 2014). Phase III studies revealed no significant difference in a composite score of death or days free from ventilation at 28 days in a population with moderate or severe ARDS (Ranieri et al., 2020).

These phase III results could be reasonably explained by patient heterogeneity, individual susceptibility to the intervention as well as drug-drug interactions in heavily-medicated critically ill patients leading to a lack of drug impact and an underpowered study with only a sub-group of patients benefitting. Receptor-mediated drug clearance, *i.e.*, clearance before IFN-beta reaches endothelial or epithelial layers, and concomitant systemic corticosteroid use influence the clinical effects seen in critically ill patients who have compromised metabolism. As will be discussed below, systemic corticosteroids impair IFN-beta induction of CD73 expression and this population with ARDS had a frequent indication for systemic steroid use (Ranieri et al., 2020).

Beyond IFN-beta-1a, the anti-inflammatory effects of some long-standing and widely used drugs are at least partially mediated by CD73. Methotrexate and sulfasalazine, used in treating rheumatoid arthritis, increase the CD73-derived production of adenosine (Morabito et al., 1998). Statins are a drug class clinically used to decrease low-density lipoprotein (LDL) formation via inhibiting 3-hydroxy-3-methylglutaryl-coenzyme A (HMG-CoA) reductase. They may also have a subtle benefit within inflammatory states through a decrease in the downregulation of CD39 and CD73 activity (Jalkanen et al., 2015; Kaneider et al., 2002). Statins prevent CD73 endocytosis, which leads to a transient increase in CD73 activity and also weakly induce CD73 synthesis (Jalkanen et al., 2015; Kiviniemi et al., 2012; Ledoux et al., 2002; Kutryb-Zajac et al., 2020). Simvastatin administration has been studied in a population with ARDS within the HARP-2 trial, and it did not improve clinical outcomes (McAuley et al., 2014). However, within *post hoc* subphenotype analysis there was a benefit of simvastatin in patients exhibiting molecularly-defined hyperinflammatory ARDS, suggesting a potential role for increased CD73 activity impacting a spectrum of this syndrome (Calfee et al., 2018).

## Potential risks of CD73-related therapy

Within animal models, concerning side effects of long-term adenosine production have been described, including fibrotic changes due to the upregulation of TGF-β and smooth muscle actin and the potential promotion of pulmonary artery hypertension (George et al., 2014; Hasan et al., 2017). These concerns may be less relevant for short-term courses targeting ARDS and other acute conditions. More acutely, adenosine signaling impacts on airway reactivity is complex with both potential beneficial effects, including bronchodilation associated with A_2A_ and A_2B_ receptor agonism, and detrimental effects, including bronchoconstriction and mucus gland hyperplasia associated with A_1_ receptor agonism (Wilson et al., 2009; Gao and Jacobson, 2017). A small trial of inhaled adenosine supplementation, due to its theoretical benefit in COVID-19 disease, identified some benefit early in the pandemic, was well tolerated without hemodynamic effects, though there was one case of moderate bronchospasm (Correale et al., 2020). This study was not further pursued, with further practical difficulty noted due to the adenosine having a half-life of less than 10 s. Historically, inhaled IFN-beta was prophylactically used to prevent asthma and chronic obstructive pulmonary disease (COPD) exacerbations (Djukanović et al., 2014). Potential mechanisms for this treatment include direct anti-viral effects and long-term protection via the induction of CD73. Reassuringly, IFN-beta has been used extensively as a long-term treatment for MS patients, with only two identified potential cases of the drug precipitating pulmonary arterial hypertension being reported within the available literature amongst the thousands of patients receiving daily IFN-beta therapy (Montani et al., 2013; Morabito et al., 1998).

## Clinical developments on platelet signaling and CD39 modulation in critical illness

Inflammation and thrombosis are tightly linked and platelets are key mediators in this process with purinergic signaling through ATP- and ADP-dependent signaling through P2X and P2Y receptors (Eltzschig et al., 2013). P2X1 is a ligand-gated ion channel receptor for ATP and following activation leads to calcium influx and potentiation of the ADP-P2Y_1_ response (Sun et al., 1998; Jones et al., 2014). Key platelet receptors for ADP include P2Y_1_ which activates phospholipase C and triggers morphologic changes associated with activation and the inhibitory G protein-coupled P2Y_12_, the most important platelet activator (Koupenova and Ravid, 2018). This has both physiologic relevance and also has been a rich target area for drug development. Inhibitors of P2Y_12_, including clopidogrel, prasugrel, and ticagrelor are frequently used as “anti-platelet drugs” to inhibit platelet function and preserve blood vessel patency in the setting of coronary artery stent placement following ischemic myocardial injury, peripheral arterial disease and ischemic stroke. Outside of these contexts, there has not been a clear application for the modulation of platelet purinergic signaling in critical illness.

Further attempts to manipulate the inflammatory pathways have aimed to target downstream enzymes by targeting eATP. This includes ongoing registered clinical trials of recombinant soluble CD39 (TIN816, Novartis Pharmaceuticals AG) for sepsis-associated AKI (NCT05996835) as well as AKI associated with major cardiac surgery (NCT05524051) with anticipated completion dates in February 2026 and September 2025 respectively; these are registered trials and do not include unpublished data from us, the authors. Others and we have considered the development of bifunctional fusion proteins of CD39 and CD73. While in the early stages of development, these recombinant fusion proteins have potential to tackle the concerns that without additional CD73 activity, excess CD39 metabolism of eATP may cause downstream AMP accumulation (Zhong et al., 2021).

## Glucocorticoids, purines and type I IFNs–the Yin and Yang in critical illness

As described, following positive phase I and II results, a phase III trial (NCT02622724, Faron Pharmaceuticals Ltd., INTEREST) was planned to investigate the role of intravenous IFN-beta in moderate and severe ARDS (Bellingan et al., 2017), yet, there was no significant difference in the 28-day composite endpoint, including mortality and the number of ventilator-free days (Ranieri et al., 2020). A *post hoc* analysis of the IFN-beta arm revealed that the simultaneous administration of systemic glucocorticoids was associated with an increase in 28-day mortality (10.6% for IFN-beta alone and 39.7% for both glucocorticoid and IFN-beta) (Jalkanen et al., 2020). Combinatorial glucocorticoid and IFN-beta use was a strong independent risk factor for death even after adjusting for disease severity. Given this concern for glucocorticoid confounding effects, IFN-beta might have continued potential for modulating inflammation in early stages of illness, particularly if there is not a clear indication for glucocorticoid use. Later it was shown that genetic susceptibility plays an important role in how a critically ill patient may react to the use of concurrent IFN-beta and glucocorticoid administration. This highlights the impact of patient heterogeneity in the ICU and the potential need for patient selection methods prior to some interventions (Jalkanen et al., 2023).

Inhaled IFN-beta-1a was examined within a phase III trial for COVID-19 infection (NCT04732949, SPRINTER). Within this population, inhaled IFN-beta did not affect the recovery time nor time to hospital discharge. This study contradicted a prior phase II trial, which had shown that inhaled IFN beta-1a increased the potential to recover two-fold relative to placebo by day 15 or 16 and three-fold by day 28 (Monk et al., 2021). Like the INTEREST trial being potentially impacted by concurrent glucocorticoid use, a potential cause for these disparate results could be the changes in care provided during the pandemic, including the appropriate widespread use of dexamethasone for patients with severe COVID-19 infections. No patients received dexamethasone in the phase II trial while 87% of patients received concurrent dexamethasone in the phase III trial (Synairgen, 2022).

A subsequent trial with intravenous IFN-beta (NCT04860518) was designed to allow for the natural sequence of IFNs and steroids to exist by treating patients with intravenous IFNs prior to entering the ICU by stopping IFNs and then initiating dexamethasone if worsening respiratory failure occurred. However, like many COVID-19 trials at this phase of the pandemic, there was a lack of eligible hospitalized patients, and the trial was halted in April 2022.

A randomized placebo-controlled phase II study (NCT03119701, INFORAAA) investigated the role of IFN-beta-1a in preventing multi-organ failure and death in patients following the open surgical repair of a ruptured abdominal aortic aneurysm. These patients faced multiple ischemic insults. First, from hypotension and decreased perfusion following the initial aneurysmal rupture, and second, from the decreased perfusion from aortic cross-clamping as part of their procedure (Makar et al., 2013; Jalkanen et al., 2016). Post-operative mortality is ∼40% and often associated with multiorgan failure (Bown et al., 2004; Biancari et al., 2011; Karthikesalingam et al., 2014).

In this post-surgical population, IFN-beta treatment on its own has shown promising results. Patients responding to IFN-beta treatment with a greater than twofold increase in their baseline CD73 levels achieved a 100% survival rate at day 30. This is a significant difference compared to the 31.6% survival of CD73 non-responders. Associated with the large mortality difference, patients with elevated levels of CD73 had reduced renal sequential organ failure assessment (SOFA) scores, an organ system-based ICU assessment score intended to predict mortality (Spicer et al., 2022). While highlighting the role of CD73 in organ protection in ischemic and acute inflammatory conditions, the small trial population may have impacted the outsized effect of CD73 induction on post-surgical mortality (Hakovirta et al., 2022).

IFN-beta has shown to be beneficial, yet the utilization of IFNs with glucocorticoids shows greater promise if following a rationale for early CD73 induction and delayed glucocorticoid administration. The interaction between IFN-beta and glucocorticoids has been described in patients receiving treatment for COVID-19 infections. Early IFN-beta treatment can lead to direct antiviral immunity and viral clearance (Lu et al., 2021; Finney et al., 2021). Dexamethasone, a glucocorticoid, was the first drug shown to reduce COVID-19 mortality, however only in a population with severe COVID-19 disease and not extending to those with mild disease (Lu et al., 2021; Horby et al., 2021; Ikeda et al., 2021). Early corticosteroid treatment may abrogate the benefit of type I IFNs and dampen natural anti-viral responses with delayed corticosteroid treatment suppressing the life-threatening cytokine storm and prevent inflammation-induced tissue damage (Cain and Cidlowski, 2020). Thus, future trials involving IFN-beta or other forms of CD73 induction should be designed to deliver initial CD73 induction prior to the potential addition of a corticosteroid.

## Conclusion

CD73 is a drug target whose activity can be modified to alleviate harmful pathophysiology precipitated by purinergic responses and inappropriately-activated immune responses (Eltzschig et al., 2013). CD73 metabolism of AMP into adenosine is protective in mouse models of hepatic, renal, and intestinal injury (Grenz et al., 2008; Hart et al., 2011; Hart et al., 2008). In early clinical data, interferon-induced CD73 expression was protective with increased catalytic activity being associated with improvements in mortality, reduced vascular leakage, and reduced secondary markers of cytokine damage (Bellingan et al., 2014; Ranieri et al., 2020; Hakovirta et al., 2022). Notably, there was an attempt to use Mupadolimab, an anti-CD73 mAb with some promising data in the context of COVID-19 infection (Miller et al., 2021; Miller et al., 2022). It is unclear whether Mupadolimab’s effects resulted from the antibody’s unique signaling through CD73 binding associated with B cell activation or a functional impact on adenosine generation. Quantitative measurements of CD73 activity may have prognostic function in identifying patients who may progress from mild sterile inflammatory states to severe inflammatory disease.

Geriatric patients often do not withstand the rigors of critical illness and the ICU. Recent work has shown that memory T cells expressing CD73 are functionally distinct and that these immune cells markedly decline with age (Fang et al., 2021). Aged patients with decreased CD73 expression on memory T cells potentially are less able to compensate for inflammatory insults. This could contribute to the increased relative mortality in critically ill patient populations (Atramont et al., 2019).

However, comparable alterations in CD39 and CD73 have been noted with extreme aging >100 years, which could have protective effects on longevity, given that peripheral blood cells in centenarians are noted to have very low CD39 and CD73 mRNA (Crooke et al., 2017). This area is further complicated as another key paper reports that ENTPD1 expression can increase markedly on activated T cells in the elderly (Fang et al., 2016). This process boosting CD39 expression could compromise vaccination efficacy and may impact T cell survival and immunoregulation. Further work is required to clarify our understanding of the dynamic changes associated with purinergic inflammation in the context of progressive, advanced aging.

The vascular endothelium is a therapeutic target in major inflammatory conditions causing critical illness (Vincent et al., 2021; Ruhl et al., 2021; Duan et al., 2017). The population of patients in the early stages of their disease course that have a potential for progression to systemic inflammatory states provide an opportunity for anti-inflammatory pre-conditioning using CD73 induction or supplementation (Bellingan et al., 2014; Ranieri et al., 2020) as well as delayed steroid administration to abrogate cytokine storm. Ongoing and proposed trials hope to assess the role of prophylactic upregulation of endothelial CD73, or alternatively CD73, and/or CD39 supplementation in surgical patients at risk of ischemia-reperfusion injury. Potential benefits and challenges of the discussed methods of modifying ATP and metabolite concentrations are briefly summarized in Table 3. Increased CD73 activity in the systemic vasculature and the hypoxia-sensitive organs may provide a greater tolerance of ischemia and intermittent poor perfusion, thereby providing a clear drug target rather than only a focus on supportive measures of care in critically ill patients.

**TABLE 3:** Summary of hypothesized advantages and disadvantages associated with methods of purinergic modification within critical illness. Selected references and/or national clinical trial ID numbers are identified; please see text for further detailed discussion.

Method	Advantage	Disadvantage	References and/or national clinical trial ID.
CD39 supplementation	Clearance of eATP with decrease in eATP-induced inflammatory signaling	Localized accumulation of AMP; without identified clinical benefit to this point	NCT05524051 NCT05996835
Alkaline phosphatase administration	Can degrade ATP to adenosine; highly active	Pluripotent effect and not specifically targeted to purinergic phosphate degradation; without identified clinical benefit to this point	Pickkers et al. (2012), Peters et al. (2015), Pickkers et al. (2018), Pickkers et al. (2024)
CD73 supplementation	Allows for adenosine-related signaling (AMP degradation); potential for greater tolerance of ischemic injury	Dependent on eATP degradation to AMP; without tested/identified clinical role	Thompson et al. (2004), Kelestemur et al. (2023)
CD39^−^CD73 supplementation	Clearance of eATP and CD39-derived AMP, allowing for adenosine-related signaling	Preclinical data only and awaiting clinical testing	Zhong et al. (2021)
CD73 induction with interferon beta 1a	Primarily induced activity within endothelial and epithelial cells; potential for greater tolerance of ischemic injury	Beneficial effect likely abrogated by concurrent steroid administration; dependent on eATP degradation to AMP; dysregulation and individual variability in IFN signaling among critically ill patients	Jalkanen et al. (2020), Hakovirta et al. (2022), Jalkanen et al. (2023) NCT02622724 NCT03119701
CD73 inhibition	Data for oncologic indication promising, particularly in combination with checkpoint inhibitors	Data for clinical benefit within oncology; benefit likely associated with the reverse (increased CD73 activity) within states of excess inflammation	Harvey et al. (2020), Roh et al. (2020) Herbst et al. (2022), Barlesi et al. (2024) NCT05221840 NCT05221840
